# Improved patient mortality predictions in emergency departments with deep learning data-synthesis and ensemble models

**DOI:** 10.1038/s41598-023-41544-0

**Published:** 2023-09-12

**Authors:** Byounghoon Son, Jinwoo Myung, Younghwan Shin, Sangdo Kim, Sung Hyun Kim, Jong-Moon Chung, Jiyoung Noh, Junho Cho, Hyun Soo Chung

**Affiliations:** 1 School of Electrical and Electronic Engineering, College of Engineering, Yonsei University, 50 Yonsei-ro, Seodaemun-gu, Seoul, 03722 South Korea; 2 Department of Emergency Medicine, College of Medicine, Yonsei University, 50 Yonsei-ro, Seodaemun-gu, Seoul, 03722 South Korea; 3 Center for Disaster Relief Training and Research, Yonsei University Severance Hospital, Seoul, 03722 South Korea

**Keywords:** Machine learning, Medical research, Prognosis

## Abstract

The triage process in emergency departments (EDs) relies on the subjective assessment of medical practitioners, making it unreliable in certain aspects. There is a need for a more accurate and objective algorithm to determine the urgency of patients. This paper explores the application of advanced data-synthesis algorithms, machine learning (ML) algorithms, and ensemble models to predict patient mortality. Patients predicted to be at risk of mortality are in a highly critical condition, signifying an urgent need for immediate medical intervention. This paper aims to determine the most effective method for predicting mortality by enhancing the F1 score while maintaining high area under the receiver operating characteristic curve (AUC) score. This study used a dataset of 7325 patients who visited the Yonsei Severance Hospital’s ED, located in Seoul, South Korea. The patients were divided into two groups: patients who deceased in the ED and patients who didn’t. Various data-synthesis techniques, such as SMOTE, ADASYN, CTGAN, TVAE, CopulaGAN, and Gaussian Copula, were deployed to generate synthetic patient data. Twenty two ML models were then utilized, including tree-based algorithms like Decision tree, AdaBoost, LightGBM, CatBoost, XGBoost, NGBoost, TabNet, which are deep neural network algorithms, and statistical algorithms such as Support Vector Machine, Logistic Regression, Random Forest, k-nearest neighbors, and Gaussian Naive Bayes, as well as Ensemble Models which use the results from the ML models. Based on 21 patient information features used in the pandemic influenza triage algorithm (PITA), the models explained previously were applied to aim for the prediction of patient mortality. In evaluating ML algorithms using an imbalanced medical dataset, conventional metrics like accuracy scores or AUC can be misleading. This paper emphasizes the importance of using the F1 score as the primary performance measure, focusing on recall and specificity in detecting patient mortality. The highest-ranked model for predicting mortality utilized the Gaussian Copula data-synthesis technique and the CatBoost classifier, achieving an AUC of 0.9731 and an F1 score of 0.7059. These findings highlight the effectiveness of machine learning algorithms and data-synthesis techniques in improving the prediction performance of mortality in EDs.

## Introduction

According to the data from the Centers for Disease Control and Prevention (CDC), there are approximately 130 million Emergency Department (ED) visits per year in the U.S., which often results in a lack of timely emergency care due to a lack of ED resources^1^. Additionally, ED crowding is a chronic and serious problem that EDs are currently facing globally, leading to a growth of negative impact on providing sufficient healthcare services^2,3^. Longer patient wait times can lead to overcrowding in the ED, resulting in a surge of patients and delays in their treatment, which is intolerable for urgent patients^4^. Especially in this era of the Severe Acute Respiratory Syndrome Coronavirus 2 (SARS-CoV-2) pandemic, this problem has become far more severe^5^. Due to a surge of patients with respiratory-related symptoms, a more specialized and efficient triage scaling system is required. In addition to the surge of ED visits, the defectiveness of the triage scaling system is also one of the main causes of ED crowding.

Most ED physicians utilize a predetermined triage scaling system specific to their country or state protocols, in an effort to reduce ED crowding. The Pandemic Influenza Triage Algorithm (PITA), Canadian Triage and Acuity Scale (CTAS), Emergency Severity Index (ESI), Manchester Triage System (MTS), and the Korean Triage and Acuity Scale (KTAS) are some of the existing ED triage scaling systems. However, the current ED triage scaling systems have certain drawbacks. They are often inaccurate due to their oversimplified decision algorithms and are time-consuming, as they rely on the practitioner’s involvement. Consequently, these systems significantly impact the waiting time of urgent patients. Moreover, the existing systems are prone to human error, as they heavily rely on subjective assessments by medical practitioners. This is widely recognized as a major contributing factor to issues, such as resource and personnel mismatches, resulting in an inefficient performance of the ED.

In recent years, there has been a growing interest in incorporating Machine Learning (ML) into ED systems, with the goal of developing ML-based triage systems. These systems aim to reduce the workload in the ED and improve the accuracy of triage decisions. ML-based triage systems have shown significant improvements compared to previous triage methods. There are two main types of ML-based triage systems. The first ML-based triage system applies ML algorithms to categorize patients into different triage levels, ranging from immediate resuscitation to discharge. The second system, which is the main focus of this paper, is an ML-based triage system that predicts patient mortality. This type of system is often referred to as an intelligent Clinical Decision Support System (CDSS). It is important to note that during the pandemic era, there were very limited medical resources and extremely high patient volume, which is why the importance of intelligent CDSSs were acknowledged more significantly. Many intelligent CDSSs aim to predict patient urgency, mortality, and ICU admission which are some of the crucial factors in EDs^6,7^. The central idea of this paper is to develop algorithms for predicting mortality, aiming to provide a more objective and rapid means of identifying the most urgent patients.

In the ED, patient data is typically organized in a tabular format. Therefore, to build an intelligent CDSS, it is advisable to use ML algorithms that excel in analyzing tabular datasets. While there are numerous ML algorithms available, determining the most effective one still remains unclear. Previous studies have explored various ML approaches, including Support Vector Machine (SVM), Random Forest (RF), and Linear Regression (LR), to predict patient urgency, mortality, and ICU admission^6,7^. Among these, LR has been frequently used. Although notable improvements have been observed with the use of these ML algorithms when compared to human-assessed triage results, there were certain limitations in these initial works. Earlier ML algorithms have exhibited sub-optimal performances, particularly in terms of Area Under the receiver operating characteristic Curve (AUC) scores. Recent ML research suggests that gradient boosting-based approaches and Neural Network (NN) algorithms may offer superior performance on tabular datasets. Additionally, existing studies have not thoroughly analyzed the imbalance within medical data, nor have they applied data-synthesis algorithms or appropriate scoring schemes to enhance prediction accuracy. Given these considerations, this paper aims to identify the most effective ML approach for predicting mortality while addressing the aforementioned limitations.

The AUC score has been commonly used as an evaluation metric for relevant algorithms. Nevertheless, as later revealed in this paper, relying solely on AUC is insufficient when evaluating an intelligent CDSS. On the other hand, the F1 score emerges as an important parameter, particularly for assessing the performance of imbalanced datasets, which is often the case in medical applications where sensitivity holds paramount importance. Additionally, data-synthesis algorithms and ensemble methods have demonstrated competence in enhancing the performance. However, the investigation of these evaluation metrics for imbalanced datasets has been overlooked by researchers, prompting the focus of this paper.

When building an intelligent CDSS, one of the major challenges faced by prediction algorithms is the need for diverse and substantial patient data, which inevitably creates privacy and data shortage issues. To address this issue, the synthesis of virtual patient data has been recognized as a practical solution. This has led to the development and application of numerous data-synthesis algorithms in the medical field. While most synthetic data has been utilized to support ML algorithms, there is an opportunity to explore more advanced combinations of data-synthesis and ML prediction technology within intelligent CDSSs. The objective of this paper is to determine the most effective combination of ML prediction and data-synthesis algorithms in predicting the mortality of patients in the ED. Furthermore, since the majority of ED patient datasets are in tabular form, this paper focuses specifically on tabular datasets and adopts twenty one features used in the PITA tabular datasets.

In summary, the main questions that will be addressed in this paper are as follows.

1. Which ML algorithm best performs in predicting mortality?
2. What kind of data-synthesis algorithms are adoptable?
3. Which combination of the ML algorithm and data-synthesis algorithm results in the best F1 score?
4. How effective are the selected twenty one features in making ML predictions?

## Methods

### Dataset

The Institutional Review Board of Yonsei University Health System Clinical Trials Center approved this study (approval number: 4-2020-0618) and informed consent was waived due to the retrospective nature of the study. All methods were performed in accordance with the relevant guidelines and regulations. Patient data were collected from the electronic medical records of 7325 individuals who sought medical attention at Yonsei Severance Hospital’s ED in Seoul, South Korea. These patients visited the hospital between January 2020 and June 2020. Yonsei Severance Hospital is designated as a COVID-19 screening clinic and is responsible for managing severe cases within its designated area. The data collection process was carried out by authorized emergency medical doctors using the hospital’s information system.

The main objective of the ML algorithms in this study was to predict the mortality of the 7325 patients. The dataset that was used for the analysis consisted of twenty one features, including gender, age, O2 apply, nebulizer usage, chest X-ray test, blood test, fluid intake, medication, high blood pressure status, diabetes mellitus (DM) diagnosis, pulmonary tuberculosis (Pul. Tbc) history, allergies, hepatitis status, other medications, mental status, systolic blood pressure (SBP), diastolic blood pressure (DBP), body temperature (BT), oxygen saturation levels (O2 Sat.), pulse rate (PR), and respiratory rate (RR). These features were routinely collected at Yonsei Severance Hospital’s ED and were originally used to calculate the Pandemic Influenza Triage Algorithm (PITA) scores. The comprehensive list of features and their corresponding data types are presented in Table 1.

**Table 1:** List of dataset features.

Features	Data type	Parameters
Gender	Boolean	$x_1$
Age	Positive integer	$x_2$
O2 Apply	Boolean	$x_3$
Nebulizer	Boolean	$x_4$
Chest X-ray	Boolean	$x_5$
Blood test	Boolean	$x_6$
Fluid	Boolean	$x_7$
Medication	Boolean	$x_8$
HiBP$^{1}$	Boolean	$x_9$
DM$^{2}$	Boolean	$x_{10}$
Pul. Tbc$^{3}$	Boolean	$x_{11}$
Allergy	Boolean	$x_{12}$
Hepatitis	Boolean	$x_{13}$
Other Medication	Boolean	$x_{14}$
Mental Status	Boolean	$x_{15}$
SBP$^{4}$	Floating-point number	$x_{16}$
DBP$^{5}$	Floating-point number	$x_{17}$
BT$^{6}$	Floating-point number	$x_{18}$
O2 Sat$^{7}$	Floating-point number	$x_{19}$
PR$^{8}$	Floating-point number	$x_{20}$
RR$^{9}$	Floating-point number	$x_{21}$

Label	Data type	Parameters
Mortality	Boolean	*y*

$^{1}$ High blood pressure $^{2}$ Diabetes mellitus $^{3}$ Pulmonary tuberculosis $^{4}$ Systolic blood pressure $^{5}$ Diastolic blood pressure $^{6}$ Blood temperature $^{7}$ O2 saturation $^{8}$ Pulse rate $^{9}$ Respiratory rate.

**Table 2:** Statistical overview of patient data (selected columns omitted).

Variables	Total (*n* = 7325)	Deceased (*n* = 99)	Not deceased (*n* = 7226)
Gender, Male (%)	44.7	58.6	44.5
Age (mean ± SD)	41.5±25.2	67.3±14.0	41.1±25.1
O2 Apply (%)	11.7	94.9	10.6
Nebulizer (%)	4.5	52.5	3.8
Chest X-ray (%)	79.9	100	79.7
Blood test (%)	49.3	100	48.6
Fluid (%)	42	98.9	41.2
Medication (%)	73.8	18.2	74.5
HiBP$^{1}$ (%)	22.2	47.4	21.8
DM$^{2}$ (%)	12.7	36.1	12.3
Pul. Tbc$^{3}$ (%)	2.2	7.2	2.2
Allergy (%)	1.3	3.1	1.3
Hepatitis (%)	2.4	6.2	2.3
Other medication (%)	26.4	61.9	25.8
Mental status, drowsy (%)	0.3	1	0.3
SBP$^{4}$ (mean±SD)	133.4±23.7	121.5±21.6	133.6±23.7
DBP$^{5}$ (mean±SD)	77.4±13.4	71.1±12.5	77.5±13.4
BT$^{6}$ (mean±SD)	37.5±0.9	37.3±1.1	37.5±0.9
O2 Sat$^{7}$ (mean±SD)	97.9±3.3	96.6±3.3	98.0±3.3
PR$^{8}$ (mean±SD)	93.8±18.4	109.3±18.7	93.5±18.3
RR$^{9}$ (mean±SD)	18.3±2.6	20.4±3.7	18.3±2.6

$^{1}$ High blood pressure $^{2}$ Diabetes mellitus $^{3}$ Pulmonary tuberculosis $^{4}$ Systolic blood pressure $^{5}$ Diastolic blood pressure $^{6}$ Blood temperature $^{7}$ O2 saturation $^{8}$ Pulse Rate $^{9}$ Respiratory rate.

### Definitions of feature variables

The study utilized twenty one specific features for analysis, namely: Gender, Age, O2 apply, Nebulizer, Chest X-ray, Blood test, Fluid, Medication, HiBP, DM, Pul. Tbc, Allergy, Hepatitis, Other Medication, Mental Status, SBP, DBP, BT, O2 Sat, PR, and RR. The statistical overview of the patient data is presented in Table 2. Six of these features, O2 Apply, Nebulizer, Chest X-ray, Blood test, Fluid, and Medication, were collected to indicate if the patient received treatment right after the initial evaluation. These features provide information on whether or not the patient was treated for particular treatment. The other six features, HiBP, DM, Pul. Tbc, Allergy, Hepatitis, and Other medication, were based on the patient’s medical history. These provide insight into the patient’s presence of high blood pressure, diabetes, pulmonary tuberculosis, allergies, hepatitis, other medication intake, and other diseases. The remaining seven features, mental status, SBP, DBP, BT, O2 Sat, PR, and RR, were measured in the initial evaluation. The patient’s mental status was categorized into alert and drowsy. The measurements included systolic and diastolic blood pressure, body temperature, oxygen saturation, pulse rate, and respiratory rate. All twenty one predictors are measured during the initial evaluation in the ED.

The O2 Apply measurement indicates whether the patient received an appropriate amount of oxygen, while the nebulizer measurement reflects the use of nebulization treatment for respiratory conditions. The chest X-ray measurement serves as a diagnostic tool for respiratory issues. The Blood test represents whether the patient underwent a complete blood count test, which helps to identify various disorders. The fluid measurement indicates whether the patient received intravenous therapy (IV) fluids during their ED visit. The Medication values indicate whether the patient received medication right after the initial evaluation.

The HiBP measurement reflects the presence of high blood pressure, and the DM determines if the patient had diabetes mellitus. The Pul. Tbc indicates the presence of pulmonary tuberculosis, and Allergy indicates any known food or medication allergies. The Hepatitis measurement reflects inflammation in the liver, and Other Medication represents the use of additional medications that may impact the patient’s current condition.

Mental Status captures the patient’s consciousness level, while SBP and DBP represent systolic and diastolic blood pressure, respectively. The BT measurement reflects the patient’s body temperature, the O2 Sat indicates the patient’s oxygen saturation level, and PR represents their pulse rate. The RR value represents the patient’s respiratory rate, measured in breaths per minute. These measurements were taken during the patient’s ED visit.

### Pre-processing

Out of the initial dataset of 7325 patients, 1543 of the patient records contained null features and were therefore excluded from the analysis. Consequently, the final input dataset consisted of 5782 patient records. To train and evaluate the machine learning (ML) system, the datasets were randomly divided into a training set (80% of the data) and a test set (20% of the data). Within the training set, a further random split was performed, creating a training subset (80% of the training set) and a validation set (20% of the training set). The size of the resulting dataset for each case are summarized in Table 3.

**Table 3:** Number of data.

Class	Mortality	
Type	True	False
Test set	19	1138
Validation set	15	910
Training set	59	3641

### Data-synthesis algorithms

The dataset used in this study, like many other medical datasets, suffers from an inherited imbalance. Specifically, there is an uneven distribution of relatively low urgent patients who were discharged after the ED visit, when compared to critically urgent patients. The deceased cases are relatively rare in the dataset. However, the primary objective of the ED is to provide timely and accurate medical care to patients with a higher probability of mortality. Therefore, it is crucial to address the imbalance and achieve an even more balanced ML training approach. To compensate for the scarcity of the deceased patient data, data-synthesis techniques including SMOTE^8^, ADASYN^9^, CTGAN^10^, TVAE^10^, and Gaussian Copula^10^ were applied to generate additional synthetic patient data.

Data-synthesis algorithms for tabular data can be classified as statistical models or deep learning models. Among deep learning approaches, Generative Adversarial Network (GAN) and Variational AutoEncoder (VAE) based methods have demonstrated successful performances in the data-synthesis domain^11^. For this paper, the statistical models SMOTE, ADASYN, and Gaussian Copula were selected as well as the GAN-based model CTGAN, and the AutoEncoder-based model TVAE.

CTGAN and TVAE, which were proposed in 2020, have gained recognition for their effectiveness in data-synthesis techniques. On the other hand, SMOTE and ADASYN are well-established statistical models that have been widely used across various domains. Additionally, the Gaussian Copula algorithm, a recently proposed model, was applied in this paper. By leveraging these data-synthesis algorithms, our goal was to generate synthetic deceased patient data and address the imbalance of the dataset. This approach ensures a more balanced representation of the deceased cases, leading to improved ML training outcomes.

### Prediction algorithms

**Figure 1:**
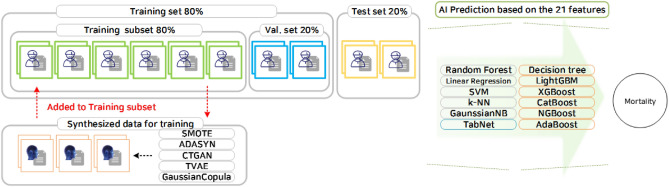
Overall prediction process.

This study utilized four different types of ML prediction algorithms: traditional machine learning, tree-based learning (with a focus on gradient boosting), and Deep Neural Network (DNN)-based learning. For traditional ML, Logistic Regression, k-Nearest Neighbors (k-NN)^12^, Support Vector Machines (SVM)^13^, Gaussian Naive Bayes (GNB), Bernoulli Naive Byes, Complement Naive Bayes, Linear Discriminant Analysis, MLP Classifier, Quadratic Discriminant Analysis, Stochastic Gradient Descent Classifier, Gradient Boosting Classifier, and Histogram-based Gradient Boosting Classification Tree were applied. In the tree-based learning category, Decision Tree^14^, Extra Tree Classifier, Random Forest^15^, AdaBoost^16^, XGBoost^17^, LightGBM^18^, CatBoost^19^, and NGBoost^20^ were included. These algorithms are known for their effectiveness in handling tabular datasets and leveraging gradient-boosting techniques. Despite the relatively underperforming nature of DNN-based learning for tabular datasets, TabNet^21^ was applied, which recently has demonstrated an exceptional performance. The overall prediction process is illustrated in Fig. 1.

### The framework of prediction models

To develop the prediction models, the data undergoes preprocessing and is subsequently divided into test, validation, and train sets. The train set is then subjected to data-synthesis algorithms, including SMOTE, ADASYN, CTGAN, TVAE, CopulaGAN, and Gaussian Copula. By applying the data-synthesis algorithm, the training set is augmented, resulting in increased sample diversity and quantity. The augmented data is then used to train multiple classification models, which consist of 22 different models. To prevent overfitting, the validation set is deployed during the training process. Once the classification models are trained, an ensemble approach is then adopted, where the predictions of each model are combined using ensemble models such as LogisticRegression, RandomForestClassifier, LightGBM, XGBoost, and CatBoost. The performance of the models are evaluated using the test set, providing a comprehensive assessment of their predictive capability. The entire framework of the prediction models is illustrated in Fig. 1.

### F1 score

Several studies have demonstrated that the evaluation of ML algorithms on imbalanced medical datasets cannot solely rely on conventional metrics like accuracy scores or AUC^22,23^. These metrics are greatly influenced by the majority class, leading to an inflated performance while the focus should be on the detection of less prevalent positive cases, which are the urgent patients in the medical domain. The urgent patients, labeled as positive cases (1), hold the highest priority in the ED, while the non-urgent patients, labeled as negative cases (0), are of secondary importance but still considered as significant. In the context of medical classification, the recall score takes the utmost importance, closely followed by the specificity score. Therefore, this paper adopts the F1 score as the primary performance measure.

## Results

The primary goal of this study is to find the best possible combination of ML classification models and data-synthesis algorithms for effectively predicting the mortality of patients visiting the ED. The main performance index is the F1 score since the F1 score is an optimal evaluation metric for an imbalanced dataset. Table 4 summarizes the top 5 results. The performance from Table 4 is calculated using only the test set.

**Table 4:** Performance of the top 5 models with the highest F1 scores for predicting mortality.

Rank	Data-synthesis	Classifier	F1	AUC	Accuracy	Precision	Recall
1	Gaussian Copula	CatBoost	0.7059	0.9731	0.9914	0.8000	0.6316
2	ADASYN	Ensembled by LogisticRegression	0.6829	0.9622	0.9888	0.6364	0.7368
3	SMOTE	Ensembled by LogisticRegression	0.6667	0.9604	0.9879	0.6087	0.7368
4	ADASYN	Ensembled by CatBoost	0.6667	0.9559	0.9896	0.7059	0.6316
5	None	Ensembled by RandomForestClassifier	0.6667	0.8930	0.9905	0.7857	0.5789

According to Table 4, the evaluation metrics of the top five models with the highest F1 scores in predicting mortality are presented. These models also went through evaluation and ranking based on their performance across multiple metrics, including AUC, F1 score, accuracy, precision, and recall. Particularly, the model in the first rank leveraged the Gaussian Copula data-synthesis technique with the combination of the CatBoost classifier. This particular model demonstrated a commendable predictive performance, performing an AUC of 0.9731 and an F1 score of 0.7059. Furthermore, it yielded an accuracy of 0.9914, followed by precision and recall values of 0.8000 and 0.6316, respectively. The model is able to predict the positive class effectively, which is the minority class in this case. The high recall is considered an important metric in this case since the goal is to effectively predict the positive class, hence providing insight to predict the urgent patients who are in need of medical attention.

Overall, the results presented in the tables indicate that various combinations of ML prediction algorithms and data-synthesis techniques were effective in predicting the mortality of patients visiting the ED. The top-ranked models exhibited strong performance, achieving high AUC and F1 scores, as well as notable accuracy, precision, and recall values. These findings highlight the potential of ML algorithms and data-synthesis methods in improving the prediction of patient outcomes in the ED settings, thereby assisting healthcare professionals in making informed decisions and providing timely and appropriate medical attention (Supplementary information 1).

## Discussion

Twenty one input features proposed in this study have demonstrated an exceptional level of performance when compared to the features introduced in other papers. These twenty one features are not only typical but also easily obtainable, making them readily applicable in other EDs. Therefore, using these twenty one features is highly recommended.

Table 5 serves as a valuable tool for the comparison of algorithms of the related studies. While it may not be entirely equitable to compare scores due to the variations in study settings and datasets, it still provides a quick overview of the efficacy of the twenty one features. In order to compare our study results to the previous works, the AUC score had to be used as an evaluation metric, since most of the previous works used the AUC to show their performance. The AUC score under 0.7 indicates poor classification, 0.7 to 0.8 indicates acceptable classification, 0.8–0.9 indicates excellent classification, and 0.9–1.0 indicates outstanding classification^24^. Based on this standard, the models presented in this paper achieved outstanding results in predicting mortality. Additionally, Miles *et al.*^25^ conducted an extensive review of 25 papers focusing on critical care patients, reporting median AUC scores of 0.89 (0.86–0.91) for neural network (NN), 0.85 (0.84–0.88) for tree-based learning, and 0.83 (0.79–0.84) for logistic regression. Notably, compared to the reported median AUC scores explained previously, this study demonstrates an improved performance in terms of the AUC score.

The model proposed in this paper not only provides a remarkable AUC score but also a sufficiently high F1 score. Even with the inherent tendency of the F1 score to be lower for imbalanced datasets, this model achieves a notably high F1 score. According to Xin Zhou et al.^26^, an artificial intelligence (AI) model can be used to make reliable predictions when the F1 score is approximately 0.7 or higher, and the AI model is expected to make very accurate predictions if the F1 score reaches 0.9 or higher. Given that the Gaussian Copula and CatBoost scheme’s F1 score for Mortality prediction surpasses 0.7, it is reasonable to conclude that this model possesses the capability to make reliable predictions.

Accordingly, the twenty one features proposed in this study exhibit exceptional predictive capabilities, while outperforming other studies in predicting mortality. Their simplicity and ease of implementation become highly suitable for adaptability in various EDs. The comparison provided in Table 5 and the findings of previous research further support the superiority of the proposed features, particularly in terms of the AUC score.

**Table 5:** Related works for comparison.

Prediction	Paper	Algorithms	AUC	Remarks
Mortality	^27^	LR$^{1}$	0.837	
Mortality	^28^	LR	0.92	
Patient urgency	^29^	DNN$^{2}$	0.86	
Patient urgency	^29^	LR	0.74	
Patient urgency	^29^	XGBoost	0.85	
Patient urgency	^29^	RF$^{3}$	0.85	
ICU admission	^30^	RF	0.88	COVID-19 patients
ICU admission	^30^	LR$^{4}$	0.86	COVID-19 patients
Mortality	^30^	RF	0.93	COVID-19 patients
Mortality	^30^	LR	0.84	COVID-19 patients
Mortality	^31^	LR	0.832	
Mortality	^31^	SVM$^{5}$	0.802	
Mortality	^31^	XGBoost	0.837	
Mortality	^32^	XGBoost	0.90	

$^{1}$ Logistic regression $^{2}$ Deep neural network $^{3}$ Random forest $^{4}$ Logistic regression $^{5}$ Support vector machine.

The predictive models used in this study outperformed the traditional triage systems, namely PITA and KTAS, that were used by the healthcare practitioners on site. In comparing the ML models with the PITA triage values, two separate values were considered, namely PITA1 and PITA2, as they were independently assigned by different practitioners for each patient. The AUC scores for PITA1 and PITA2 were 0.841 and 0.695. With the ML models achieving AUC scores above 0.9, it can be confidently concluded that they outperformed the traditional triage system. The implementation of intelligent CDSS can significantly assist practitioners in making informed decisions during treatment and examination. The subjectivity of medical practitioners contributed to the inconsistencies observed between PITA1 and PITA2, underlining the need and importance of intelligent CDSS in healthcare settings.

The data-synthesis algorithm proposed in this study was effective in generating synthetic data that were similar to the original data. The synthetic data were used to train the ML models, which exhibited strong performance in predicting patient mortality. The results of the experiments demonstrate that the synthetic data were effective in training the ML models, achieving high F1 scores. For example, the top-ranked model in terms of F1 score for predicting patient mortality used the Gaussian Copula data-synthesis algorithm to augment the data. When compared to the same classifier but excluding the data-synthesis algorithm, the F1 score increased from 0.5333 to 0.7059. The results of the experiments demonstrated that the synthetic data were effective in training the ML models, achieving higher AUC scores and F1 scores.

## Conclusion

In this study, a novel approach to predicting patient mortality in EDs using ML algorithms and data-synthesis techniques is proposed. The methodology was evaluated using a dataset of 7325 patients who visited the ED of Severance Hospital in Seoul, South Korea. The results demonstrate that the proposed techniques were effective in predicting the mortality of patients visiting the ED. The models exhibited a strong performance, achieving high AUC and F1 scores, as well as accuracy, precision, and recall values. These findings accentuate the potential of ML algorithms and data-synthesis methods in improving the prediction of patient outcomes in ED settings, thereby assisting healthcare professionals to perform informed decisions and provide more timely and appropriate medical care.

### Supplementary Information

Supplementary Information.

## Data Availability

The datasets generated and/or analyzed during the current study are not publicly available since they contain information that may compromise the privacy of the patients. Data is however available from the corresponding author upon reasonable request and with permission of the Yonsei University College of Medicine. The trained models and code to run predictions are publicly available on Github (https://github.com/sonhun99/ED_Urgency).
